# Comparison between rigid telescopic and flexible fiberoptic laryngostroboscopy^☆^^☆☆^

**DOI:** 10.1016/j.bjorl.2025.101599

**Published:** 2025-04-09

**Authors:** Rogério Aparecido Dedivitis, Mario Augusto Ferrari Castro, André Vicente Guimarães, Caio Paschoalin Trindade

**Affiliations:** a Universidade de São Paulo (USP), Faculdade de Medicina, Hospital das Clínicas, Departamento de Cirurgia de Cabeça e Pescoço, São Paulo, SP, Brazil; b Universidade Metropolitana de Santos, Santos, SP, Brazil; c Centro Universitário Lusíada, Departamento de Cirurgia, Santos, SP, Brazil; d Universidade São Judas Tadeu, Cubatão, SP, Brazil

**Keywords:** Laryngoscopy, Stroboscopy, Voice disorders, Parkinson disease, Phonation, Vibration, Vocal cords, Fiber optic technology, Dysphonia

## Abstract

**Objective:**

Stroboscopy can uncover significant laryngeal abnormalities in patients with Parkinson’s Disease (PD). Rigid telescope and flexible fiberscopy present differing advantages. Objective: To compare the stroboscopic findings observed using rigid telescopy to those obtained through fiberoptic examination.

**Methods:**

A prospective study was conducted in order to evaluate 36 patients with PD from January 2018 to December 2019. The HY – Degree of Disability Scale was adopted in order to assess individual patients’ levels of impairment. The patients included in this study were grouped- higher than 1.5 on the scale. There were 22 men and 14 women, with ages varying from 41 to 78. Three observers analyzed the recording data, with a protocol for stroboscopic evaluation being adopted.

**Results:**

Tremor, open phase closure and vocal fold bowing were the most common findings among patients. Aperiodic voice in 4 cases recommended against stroboscopic analysis. Strong gag reflex in another 3 cases, made evaluation with rigid telescope impossible. The irregularity of the edge, glottic closure, prevalence of the glottic cycle phase, amplitude; mucosal wave; vibratory behavior; phase symmetry, periodicity and movement extension were evaluated by both methods. The vibratory source was exclusively glottic in all cases.

**Conclusion:**

Videolaryngostroboscopy can be performed by means of both methods – rigid and fiberoptic examination.

**Level of evidence:**

Level III.

## Highlights

• The use of rigid and fiberoptic telescopic laryngostroboscopy is well established and each one presents advantages.
• Seventy to 92% of patients with Parkinson's Disease present tongue, larynx, and pharynx disorders.
• Patients unable to maintain regular voice production under the rigid approach can be evaluated using fiberoptic stroboscopy.
• When both methods are technically feasible, the rigid telescope approach is preferrable due to providing greater anatomical detail.

## Introduction

The utility of videostroboscopy for clinical evaluation of the larynx is well established.^1^ This technique utilizes intermittent light pulses that illuminate the vocal folds and whose fusion by the human eye results in mucosal motion. Combining the stroboscope and the laryngoscope gives an excellent slow- motion representation of vocal fold movement.^2^

Parkinson’s Disease (PD) is a chronic, progressive condition with slowly developing symptoms.^3^ The characteristic symptoms, known as cardinal, are tremors, muscle rigidity, akinesia, bradykinesia and postural changes, with occurrence and intensity varying from patient to patient. Voice and speech alterations are included in these symptoms and are frequent disorders which respond poorly to clinical treatment. Seventy to 92% of patients present tongue, larynx and pharynx disorders.^4^ Glottic resistance, air flow increase, low subglottic pressure and decreased loudness are often found in PD.^5^ The vocal changes can be assigned to the incomplete glottic closure, reduction in synergy, laryngeal muscle activation, muscle atrophy or fatigue, vocal fold tension or motion asymmetry and vocal fold or respiratory muscle rigidity.^6^ Furthermore, as a consequence of the disease, the vocal tract requires greater constriction to produce some phonemes. Difficulty in articulation is a frequent symptom present during all phases of the disease. However, it is more debilitating in advanced stages, constituting an intrusive symptom in patients who are voice users.^4^ The monotone voice is characterized by homogenous phrase production, with pauses, and loss of intonation and natural cadence.^7^ Stroboscopy can uncover significant laryngeal abnormalities, such as abnormal adduction and abduction, bilateral vocal fold atrophy and phase asymmetry.^8^

The rigid telescope is supposed to provide more anatomical detail of the vocal folds than the fiberoptic endoscope. Thus, in spite of the gag reflex presented by some patients and the non-physiological conditions of the examination, the telescope enables stroboscopic evaluation in cases of dysphonia.^9^ On the other hand, body posture during examination with a rigid telescope can interfere with glottic vibration. Flexible fiberscopy allowed the subject to phonate in the same position he/she would during natural speech.^10^ Thus, the advantages of the strobofiberscopic over the strobotelescopic video system are: a larger pool of potential subjects; and patients can phonate while maintaining normal head position during examination.^11^

The aim of this study is to compare the stroboscopic findings observed using rigid telescopy to those obtained during fiberoptic examination of the same patients, under similar conditions in a routine clinical situation. A with a PD patient population, which was chosen since stroboscopic laryngeal abnormalities are usual among them.

## Materials and methods

This study was approved by the institutional Review Board of Centro Universitário Lusíada, under the number 120/2011.

Thirty-six PD patients were prospectively evaluated from January 2018 to December 2019. They were recruited from the institute’s Neurology Service where the study was carried out. Patients presenting the following criteria were excluded from this study: prior stroke, severe cranial trauma or encephalitis, previous treatment with neuroleptics, spontaneous symptom remission, unilateral clinical symptoms for 3-years, ocular supranuclear paralysis, cerebellar signs, early autonomic signs, pyramidal liberation with Babinski’s signal, presence of brain tumor or communicating hydrocephalus, negative response to high doses of levodopa or explosion to metilpheniltetraperidinium. The HY – Degree of Disability Scale was adopted to determine individual patients’ degree of impairment.^12^ It comprises 5 classification stages that evaluate PD severity. The patients included in this study presented a score higher than 1.5. As such, stage I patients were excluded, since they do not present voice or speech symptoms. One patient was excluded due to dementia since he was not able to undergo the proper evaluation.

There were 22 men and 14 women, whose ages varied from 41 to 78. The duration of clinical complaints varied from 3-months to 22-years. All patients were undergoing PD drug treatment. The disease stage varied from 1.5 to 5, according to the HY – Degree of Disability Scale^12^: stage 1.5–8 (21.05%); stage 2.0–6 (15.8%); stage 2.5–10 (26.31%); stage 3.0–8 (21.05%); stage 4.0 – 2 (5.26%); and stage 5.0 – 2 (5.26%) – Table 1.

**Table 1:** Patient characteristics (n = 36).

Age (years)	Range	41‒82
	Median	70.0
	Average (SD)	68.2 (9.9)
Time of disease (years)	Range	1 ‒ 22
	Median	5.0
	Average (SD)	6.2 (5.2)
Hoen Yahr Index	Range	1.5 ‒ 5.0
	Median	2.5
	Average (SD)	2.5 (0.9)

SD, Standard Deviation.

Videolaryngostroboscopy was performed with a rigid 70 ° Karl Stroz® telescope connected to a Kay RLS 9100 B Laryngeal Stroboscope light source. The fiberoptic evaluation used a Xion® nasopharyngoscope. Images were viewed on a Sony KV-1311 CR monitor and recorded onto a Sony SLV-60HFBR VHS tape recorder. Patients were asked to maintain production of the vowels /e/ and /i/. The video recordings were viewed and rated simultaneously in a nonblinded manner by 3 authors of this study experienced in laryngostroboscopy. Qualitative assessment and subjective ratings were discussed in order to reach a consensus. The protocol for stroboscopic evaluation considered the following aspects: free board rightness; glottic closure; prevalence of the glottic cycle phase; amplitude; mucosal wave; vibratory behavior; phase symmetry; periodicity; movement extension; and source of vibration.^13^

## Results

The laryngostroboscopic findings are presented in Table 2. Tremors were the commonest finding. In 4 cases of significant tremor, the voices were not periodic enough to allow stroboscopic evaluation. Another 3 cases could only be evaluated using fiberoptic laryngoscopy because the patients presented significant gag reflex response during the rigid telescopic evaluation, even after applying topical anesthetic spray. We considered their voices irregular under periodicity evaluation.

**Table 2:** Stroboscopic findings.

**Aspect**		**Evaluation**				
**Free heard rightness**	**Straight**	**Slightly irregular**		**Moderately irregular**		**Severely irregular**
Rigid	34	2		0		0
Fiberoptic	34	2		0		0
**Glottic closure**	**Complete**	**Vocal fold bowing**		**Posterior triangular chink**		
Rigid	20	12		4		
Fiberoptic	20	12		4		
**Prevalence of the glottic cycle phase**	**Opened**	**Normal**		**Closed**		**NA**
Rigid	20	9		0		7
Fiberoptic	21	11		0		4
**Amplitude**	**Normal**	**↓**	**↓↓**	**↓↓↓**	**Absent**	**NA**
Rigid	24	3	2	0	0	7
Fiberoptic	26	4	2	0	0	4
**Mucosal wave**	**Normal**	**↓**	**↓↓**	**↓↓↓**	**Absent**	**NA**
Rigid	26	3	0	0	0	7
Fiberoptic	28	4	0	0	0	4
**Vibratory behavior**	**Always total presence**	**Occasional total presence**		**Always total absence**		**NA**
Rigid	27	2		0		7
Fiberoptic	31	1		0		4
**Phase symmetry**	**Regular**	**Generally regular**		**Generally irregular**		**NA**
Rigid	27	2		0		7
Fiberoptic	31	1		0		4
**Periodicity**	**Regular**	**Generally regular**		**Generally irregular**		**NA**
Rigid	29	0		0		7
Fiberoptic	32	0		0		4
**Movement extension**	**Similar**	**Right > left**		**Left > right**		**NA**
Rigid	29	0		0		7
Fiberoptic	32	0		0		4
**Vibratory source**	**Glottic**	**Supraglottic**		**Mixed**		
Rigid	36	0		0		
Fiberoptic	36	0		0		

NA, Not Available.

The free board rightness and the glottis closure were evaluated by both methods. Even among patients with vocal tremor and aperiodic voices, those aspects could be verified. However, the other aspects could not be studied in the patients with aperiodic voice in either method nor in the patients with gag reflex by rigid laryngoscopy, due to the lack of regular voice production over a long enough period.

After the vocal tremors, open phase closure and vocal fold bowing were the most commonly observed symptoms among patients. The vibratory source was exclusively glottic in all cases. Even among patients with aperiodic voices, the vibratory source could be easily verified in the glottis edge. All posterior triangular chinks were detected in women and considered a physiological finding.

## Discussion

The human vocal folds present a wide range of sound production, which can be attributed to extremely precise neuromuscular control and significant flexibility of the structures due to specific histological characteristics.^14^, ^15^

In PD, glottic resistance decreases, air flow increases, and subglottic pressure and vocal intensity diminish.^5^ Vocal changes can be attributed to incomplete glottic closure and synergy, reduced laryngeal muscle activation, muscle atrophy or fatigue, vocal fold tension or movement asymmetry, and vocal or respiratory muscle fold rigidity.^6^, ^16^

Videolaryngoscopy is a useful and effective assessment and documentation method for physiological and pathological conditions of the larynx. It is of great value for making accurate diagnoses and planning adequate treatment. It allows instant and simultaneous voice and video recording and subsequent analysis. Videolaryngoscopy can be accomplished with either a flexible fiberscope or a rigid right-angled telescope. Fiberscopic videolaryngoscopy is more useful for voice analysis of speech disorders and evaluation of laryngeal functions such as phonation, singing and swallowing. Telescopic videolaryngoscopy is superior for critical evaluation of anatomical and pathological changes of the laryngeal structures as well as close-up examination of vocal fold function. While fiberscopic laryngoscopy is technically easy, fiberscopic video documentation is much more difficult than telescopic video documentation. Telescopic videolaryngoscopy provides clearer and sharper images of the larynx.^17^ They should be considered complementary methods.^9^

Since rigid lryngoscopy alters the normal phonatory anatomy, flexible laryngoscopy is commonly viewed as better suited to evaluating the neurological integrity of the larynx.^18^ The examiner’s familiarity and experience with endoscopy may also influence the information obtained from the assessment, suggesting that training with equipment is necessary.^19^ Stroboscopy can change or modify the diagnosis in 10%–47% of cases, however, it may be underused. Expense, access, expertise and perceived need may have limited its use. Specialists may not appreciate the differences between laryngoscopy and stroboscopy. They are less comfortable diagnosing neurological disorders than those associated with structural laryngeal abnormalities.^20^

Tremors were the main reason for the aperiodic voices observed in our study. The lack of periodicity impedes the realization of a full stroboscopic analysis. Gag reflex was another impediment, but only for the rigid telescope examination. Thus, it is clear that for selected cases in which the patient is unable to maintain regular voice production under the rigid approach, the fiberoptic method is preferrable to achieve a complete stroboscopic evaluation.

The evaluation of the free board rightness and glottis closure is mainly morphological. As a result, even in those patients with aperiodic voices or strong gag reflex reactions, both methods – rigid and fiberoptic – can efficiently assess those aspects. On the other hand, the aspects dependent on having enough periodic voice production for evaluation cannot be determined unless the assessment can be technically performed. The vibratory source was exclusively glottic in all cases. There was a lack of vibration of the supraglottic structures, even with aperiodic samples, where vibration can still be realized, but in an irregular fashion.

An incomplete closure of the posterior part of the glottis may be the case in normal individuals without voice disorders. Open posterior chink during phonation has been observed in normal females regardless examination device – rigid or fiberoptic. On the other hand, in normal males, complete vocal fold closure is the most common finding, however, incomplete posterior closure also occurs, mainly in soft phonation.^10^

## Conclusion

Videolaryngostroboscopy can be performed by means of both methods – rigid and fiberoptic examination. Patients unable to maintain regular voice production under the rigid approach can be evaluated using fiberoptic stroboscopic evaluation. When both methods are available and technically feasible, the rigid telescope approach is preferrable due to providing greater anatomical detail.

## Conflicts of interest

The authors declare no conflicts of interest.

## Meeting of ethical standards

O presente artigo faz parte da coleção de artigos que anteriormente pertenciam a Sociedade de Cabeça e Pescoço (SCCP) e foram cedidos ao Brazilian Jornal of Otorhinolaryngology (BJORL).
